# MLL-Rearranged Leukemias—An Update on Science and Clinical Approaches

**DOI:** 10.3389/fped.2017.00004

**Published:** 2017-02-09

**Authors:** Amanda C. Winters, Kathrin M. Bernt

**Affiliations:** ^1^Division of Pediatric Hematology/Oncology/BMT, University of Colorado School of Medicine and Children’s Hospital Colorado, Aurora, CO, USA

**Keywords:** mixed lineage leukemia, infant leukemia, epigenetics, targeted inhibitor, chemotherapy, HSCT

## Abstract

The mixed-lineage leukemia 1 (MLL1) gene (now renamed *Lysine [K]-specific MethylTransferase 2A* or *KMT2A*) on chromosome 11q23 is disrupted in a unique group of acute leukemias. More than 80 different partner genes in these fusions have been described, although the majority of leukemias result from *MLL1* fusions with one of about six common partner genes. Approximately 10% of all leukemias harbor *MLL1* translocations. Of these, two patient populations comprise the majority of cases: patients younger than 1 year of age at diagnosis (primarily acute lymphoblastic leukemias) and young- to-middle-aged adults (primarily acute myeloid leukemias). A much rarer subgroup of patients with *MLL1* rearrangements develop leukemia that is attributable to prior treatment with certain chemotherapeutic agents—so-called therapy-related leukemias. In general, outcomes for all of these patients remain poor when compared to patients with non-*MLL1* rearranged leukemias. In this review, we will discuss the normal biological roles of MLL1 and its fusion partners, how these roles are hypothesized to be dysregulated in the context of *MLL1* rearrangements, and the clinical manifestations of this group of leukemias. We will go on to discuss the progress in clinical management and promising new avenues of research, which may lead to more effective targeted therapies for affected patients.

## Structure and Function of Wild-Type MLL1

### Mixed-Lineage Leukemia 1 (MLL1) Protein Structure and Binding Partners

**Figure 1:**
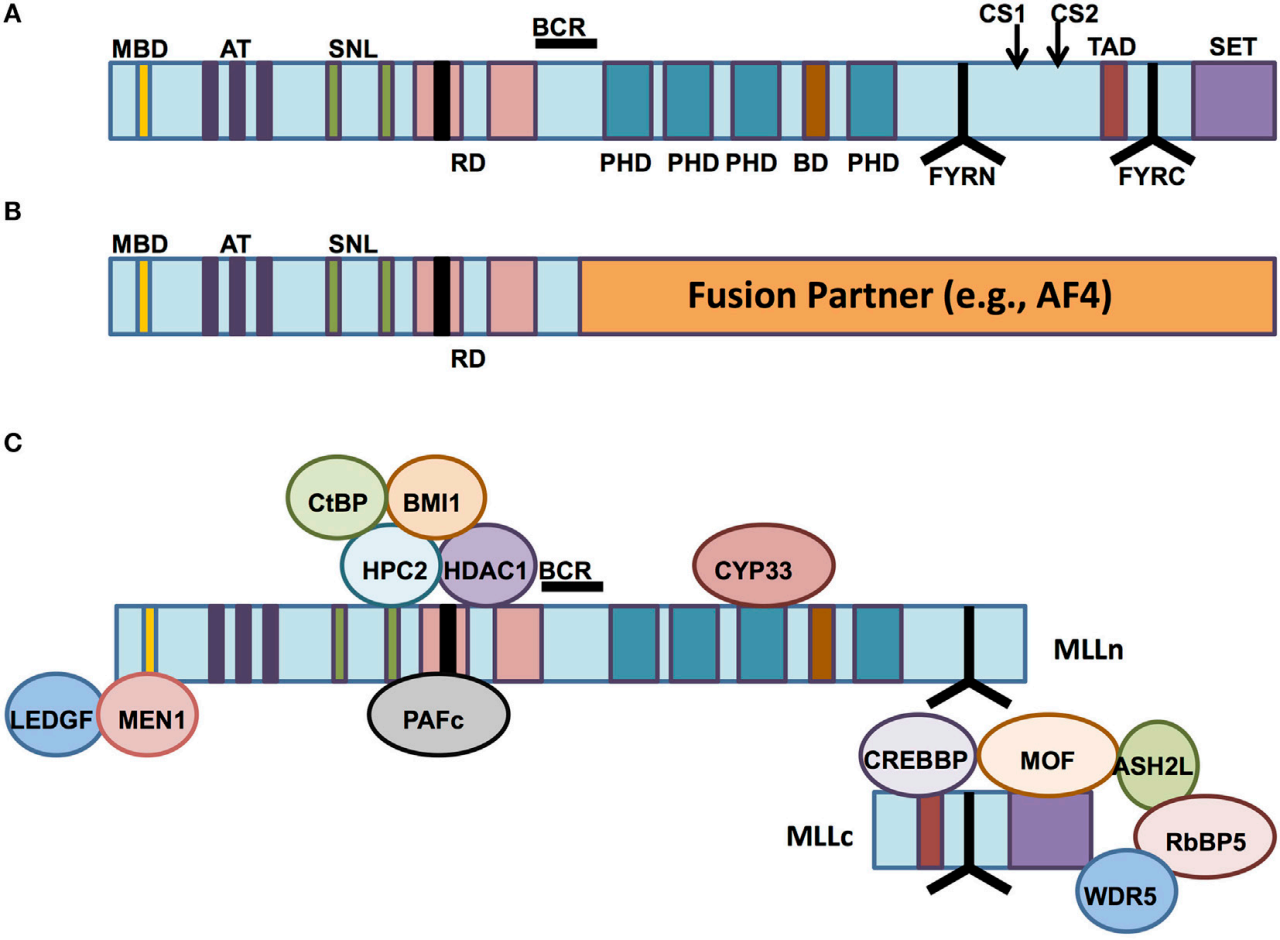
**The structure of mixed-lineage leukemia (MLL) and normal vs aberrant MLL complexes**. **(A)** The structure of the wild-type MLL protein, emphasizing the functional domains. MBD, Menin-binding domain; AT, AT hooks; SNL, speckled nuclear localization domains; RD, repression domains (black box in first RD represents the CXXC domain); BCR, breakpoint cluster region; PHD, PHD fingers; BD, bromodomain. CS1 and CS2 are the taspase-1 cleavage sites, and FYRN and FYRC are the domains whereby MLL-N and MLL-C interact after cleavage. TAD, transactivation domain; SET, H3K4 histone methyltransferase domain. **(B)** MLL fusion proteins are caused by chromosomal rearrangements leading to in-frame fusions between *N*-terminal MLL (to the BCR) and any of 80 different fusion partners. PHD domains, transactivation domains, and the SET domain are lost. **(C)** MLL-interacting proteins. Proteins involved in repressive functions of MLL are grouped above the MLL protein (regulated by CYP33), whereas proteins involved in activation of MLL-dependent transcription are grouped below the MLL protein schematic.

The normal *MLL1* gene at the 11q23 locus encodes an approximately 500-kDa nuclear protein with multiple functional domains and binding partners (Figure 1A), whose structure was first described by both Tkachuk et al. and Gu et al. (1, 2) and which is expressed in a wide variety of normal human tissues (3). The *N*-terminal portion of the protein contains a domain for binding Menin, a protein that serves as a link between MLL1 and the chromatin-binding protein lens epithelium-derived growth factor (LEDGF). LEDGF is a binder of dimethylated H3K36 (placed by ASH1L). The association of MLL1 with Menin/LEDGF is particularly critical for the function of MLL fusions, but also affects wild-type MLL1 (4–10). The *N*-terminus also contains AT-hook motifs (DNA-binding domains), speckled nuclear localization domains 1 and 2 (SNL-1 and SNL-2), and two repression domains (RD1 and RD2), the first of which (RD1) also contains a CxxC domain (1, 11–13). The CxxC domain has homology to DNA methyltransferase 1 (DNMT1), which methylates cytosine residues of DNA (11, 14). Although DNMT1 preferentially targets hemimethylated CpG motifs, the MLL1 CxxC domain binds non-methylated CpG DNA (15). All of these domains are typically conserved in chimeric MLL1 fusion proteins (12). The middle portion of MLL1 contains four plant homeodomain (PHD) fingers (which mediate protein–protein interactions) and a bromodomain (which mediates binding to histones with acetylated lysine residues). The *C*-terminal portion contains a transcriptional activation domain and a SET domain (1, 12, 16). The third PHD finger allows association between MLL1 and the cyclophilin CYP33, which is important for negative regulation of certain MLL1 target genes (17). The SET (Su(var)3-9, enhancer of zeste, trithorax) domain is homologous to that of *Drosophila* trithorax and catalyzes mono-, di-, and trimethylation of lysine 4 on histone 3 (H3K4) *in vitro* (1, 18). These latter four domains (PHD finger, bromodomain, activation domain, and SET domain) are all lost in most MLL1 fusion proteins (12) (Figure 1B).

After its translation, wild-type MLL1 is proteolytically cleaved by the enzyme taspase-1 (19, 20). The resulting 320-kDa *N*-terminal fragment (MLL-N) contains all domains except the transcriptional activation domain and the SET domain, both of which are retained by the 180-kDa *C*-terminal fragment (MLL-C). MLL-N and MLL-C normally associate with one another as components of a multiprotein complex that regulates chromatin modification and gene expression (19, 21) (Figure 1C). Other essential proteins that make up the core of the MLL1 complex include RbBP5, Ash2L, and WDR5 (21). These three proteins form a complex that is able to bind a variety of H3K4 methytransferases with SET domains, including MLL1. Recent biochemical and structural analyses of the interactions between the complex members reveal that the RbBP5-Ash2L heterodimer interaction with MLL1 stabilizes it in the catalytic conformation, whereas WDR5 acts as a bridge between the RbBP5-Ash2L complex and MLL1 itself (22). The WDR5 bridge is not needed for the interaction between RbBP5-Ash2L and other MLL family members, but it is essential for the H3K4 methyltransferase activity of MLL1. MLL1 recruits these other components, along with other chromatin remodeling proteins such as the histone acetyltransferases CBP/p300 and hMOF, to specific target genes (21, 23, 24). In fact, recruitment of these other histone-modifying proteins, particularly hMOF, has recently been shown to be crucial for MLL1 target gene expression, whereas the H3K4 methyltransferase activity of MLL1 is dispensable in this regard (24).

The *N*-terminal portion of MLL1 present in translocation-encoded fusions loses its ability to interact with MLL-C (19). The functional consequence of this feature is not clear. In most leukemias, residual core complex including MLL-C would be expected to be present and retain its histone methyltransferase activity, either from expression of the reciprocal fusion (although this probably happens only in a minority of patients) or from the second, non-rearranged *MLL1* allele. There is debate whether the second allele is required—on one hand, experimental data from knockout mice suggest that it might be (25, 26), on the other hand, deletion of the second *MLL1* allele has been reported in patients (27) and also occurs in the ML2 cell line. Whether leukemias with deletions of the MLL1 wild-type allele retain residual wild-type function through expression and cleavage of a reciprocal fusion is unclear, as is the role of the reciprocal fusion in general. Wilkinson et al. reported that the MLL-AF4 fusion activates expression of *RUNX1* and that the RUNX1 protein then interacts with the AF4-MLL reciprocal fusion and the MLL-C complex proteins (28). The authors hypothesized that interaction of AF4-MLL enhances its coactivation of RUNX1 target genes, although they were not able to successfully target AF4-MLL *via* siRNA for functional confirmation of this theory. Furthermore, a reciprocal translocation predicted to result in the *expression* of a reciprocal fusion transcript was found in only 24 of 182 *MLL-*rearranged (*MLL-r*) patients (29). The fact that in most patients the reciprocal fusion is likely not expressed strongly argues against a critical role.

### Physiologic Functions of MLL1

MLL1 is both structurally and functionally homologous to the *Drosophila melanogaster* protein trithorax (1), which is involved in epigenetic regulation of defined developmental genes [reviewed in Ref. (30)]. Homozygous deletion of *Mll1* in murine embryos results in lethality at E10.5–E12.5, with null embryos showing abnormal facial development and innervation of embryonic structures, as well as abnormal fetal hematopoiesis (31–33). *Mll1* ± (heterozygous) embryos display both body segmentation abnormalities and decreased numbers of cells of several hematopoietic lineages. Many of these defects closely resemble those seen upon knockout of developmental patterning genes, such as the *homeobox* (*Hox*) genes, many of which (*Hoxa9, Hoxa7*, and *Hoxc8*) have been identified as Mll1 target genes. Although Hox genes are expressed in *Mll1*^−^*^/^*^−^ embryos before the E9.0 stage, their expression is not maintained at later time points in the absence of Mll1 (34). These findings indicate that Mll1 is required for the maintenance, and not the initiation, of Hox gene expression. In steady-state adult murine hematopoiesis, hematopoiesis-specific knockout of *Mll1* resulted in moderate to severe impairment of stem cell function (35, 36).

Identification of MLL1 target genes involved in embryogenesis and hematopoiesis has been the goal of multiple studies. MLL1 has been reported to occupy as much as 5,000 genes in leukemia cell lines and cultured lymphoblasts (37) and a smaller number of genes in fibroblasts (38). MLL1 binding correlated with the presence of H3K4me3 and occupancy of RNA polymerase II, suggesting that despite the presence of multiple negative regulatory domains in the MLL1 protein, the net outcome of MLL1 binding is typically transcriptional activation. Despite correlation of MLL1 binding with H3K4 trimethylation, MLL1 is not the methyltransferase responsible for the deposition of the majority of H3K4 trimethylation in any tissue examined to date, as knockout does not result in decreased global levels of H3K4me3 (24).

## Normal Functions of the Common MLL1 Fusion Partners

Leukemia-associated translocations involving 11q23 have been shown to generate in-frame fusions of the *MLL1* gene to more than 80 different partner genes (29). *N*-terminally truncated *MLL1* alone is not sufficient to transform cells (39, 40). This finding argues for a crucial contribution on the part of the fusion partner proteins to leukemogenesis. Although the proteins encoded by the 80 + *MLL1* partner genes seemingly have diverse structures and functions, two common features have emerged that likely have importance for the oncogenic potential of the chimeric protein. First, many of the partners, including the ones most frequently encountered in MLL1 fusions, are nuclear proteins involved in the regulation of transcriptional elongation [though interaction with the positive transcription elongation factor b (pTEFb) complex and phosphorylation of Pol II] and direct or indirect recruitment of the H3K79 histone methyltransferase DOT1L (41–49). Second, many partners, including those that are cytoplasmic, have been shown to form complexes in the nucleus as fusion proteins (50). A revealing study demonstrated that a fusion construct of *Mll* exons 1–8 and *lacZ*, the gene encoding the non-oncogenic enzyme β-galactosidase, was able to cause leukemias in mice, albeit with longer latency and lower incidence than the more traditional *Mll-Af9* fusion (51). Importantly, the formation of a tetramer is essential for the functionality of the enzyme β-galactosidase, and all of the leukemia cells demonstrated β-galactosidase activity, suggesting that the fusion proteins also oligomerized. Martin et al. confirmed that dimerization of MLL1 is transforming through the fusion of MLL1 to FKBP12, an inducible dimerizer (52). A final interesting consideration is that *N*-terminal MLL1 is normally destabilized by the loss of interaction with MLL-C (19). The MLL1 fusion construct loses the domain necessary for MLL-C binding and therefore would be expected to be degraded—since it is not, the fusion partner may also play a role in enhancing the stability of the fusion protein.

### The AF4 Protein Family

The AF4 protein (ALL1-fused gene from chromosome 4) is fused in-frame to MLL1 as a result of a t(4,11)(q21,q23) translocation (2). This fusion is responsible for approximately 50% of cases of infant acute lymphoblastic leukemia (ALL) with *MLL1* rearrangement and more than 75% of adult *MLL-r* ALLs (29). AF4 is a member of the ALF (AF4, LAF-4, FMR-2) family of nuclear proteins (41, 42). Two additional family members (LAF-4 and AF5q31) have been identified in MLL1 fusions from patient samples (53, 54). These proteins share several regions of homology, including a region rich in serine residues that has been shown to have transactivation properties in reporter assays and which is conserved in fusions with MLL1 (55, 56). The functions of the known family members remain incompletely characterized. However, AF4 knockout mice display significant delays in lymphopoiesis (generation of B and T cells) (57). AF4 has been shown to interact with pTEFb and DOT1L (44–46). pTEFb is a complex of cyclin T1/T2 and cyclin-dependent kinase 9 (CDK9), which phosphorylates the *C*-terminal domain of RNA polymerase II and thus promotes transcriptional elongation (58). AF4 binding to pTEFb enhances PolII-CTD phosphorylation and promotes gene transcription. AF4 family members may also interact with another transcriptional complex, selectivity factor 1 (SL1), which is composed of TATA-binding protein and four associated factors, and this association may play a role in direct recruitment of RNA polymerase II to target genes (59).

### AF9 and ENL

*ALL1-fused gene from chromosome 9 (AF9)* and *eleven-nineteen leukemia (ENL*) are the second and third most common fusion partners of *MLL1*, and these fusions arise from the t(9,11)(p22,q23) and t(11,19)(q23,p13.3) translocations, respectively (29). *MLL-AF9* is most commonly associated with myeloid leukemias, while *MLL-ENL* is prevalent in both lymphoid and myeloid leukemias (60). AF9 and ENL have highly similar structures. Both proteins have a conserved *C*-terminal coiled coil region with transactivation properties that is necessary and sufficient for leukemic transformation in the context of *MLL1* fusions (40). Furthermore, AF9 and ENL have also been shown to interact with AF4 *via* their *C*-termini and thus be part of AF4 containing complexes that also bind pTEFb and DOT1L (44–49). The *C*-terminal domains mediating this interaction are conserved in MLL1 fusions (43) and mutation of the DOT1L-binding domain of ENL in MLL-ENL cells abrogated colony formation and reduced Hox gene expression typically associated with transformation (45).

Similar to MLL1, AF9 and ENL have roles in the epigenetic/transcriptional control of developmental pathways (31, 61). Wild-type AF9 in mice and humans seems to have a regulatory function specifically in megakaryocyte/erythrocyte lineages (61, 62). AF9 and ENL have been shown to interact with the protein Polycomb 3, also known as CBX8, a component of polycomb repressive complex 1 (PRC1), which is implicated in maintenance of stable repression of genes, and with certain isoforms of the BCL-6 corepressor (45, 63–65). However, rather than mediating transcriptional repression, the role of CBX8 in the context of MLL fusions appears to mediate the recruitment of the histone acetyl transferase Tip60, thereby promoting fusion target gene expression (66). Finally, the *N*-terminal YEATS domain of ENL and AF9 have reader function recognizing histone proteins 1 and 3 (H1 and H3) acetylation and, as recently demonstrated, crotonylation (49, 67–69). The wild-type AF9 YEATS domain has been reported to be critically involved in the recruitment of DOT1L to chromatin and H3K79 methylation-mediated transcriptional control [(49) and see “DOT1L Inhibitors” section]; however, in the MLL1 fusion, the YEATS domain is typically excluded (40). It is possible that the *N*-terminal MLL1 fragment supplies this function; however, this has been difficult to experimentally confirm. The precise function of these various binding partners to the function of AF9 or ENL in their wild-type or MLL1-fused states will still require more investigation.

### AF10 and AF17

AF10 was the first MLL1 fusion partner to be shown to interact with DOT1L (70). AF10 and the structurally related AF17 (also a rare fusion partner) are consistently co-purified with DOT1L and part of the canonical DOT-complex (47). AF10 is required for di- and tri- (although not mono-) methylation of H3K79 by DOT1L (71). The PHD finger of AF10 specifically binds to unmodified H3K27 (72). Although both AF10/AF17 and AF9/ENL co-purify with DOT1L, it is unclear whether all these proteins reside in one or in two separate complexes and what the relationship of these complexes is to elongation complexes containing AF4, AF5, and pTEFb or SL1 (46–48, 59).

## Transcriptional Dysregulation in the Context of MLL1 Fusions

### Controversies Around and Potential Roles of an Oncogenic Multiprotein Complex

**Figure 2:**
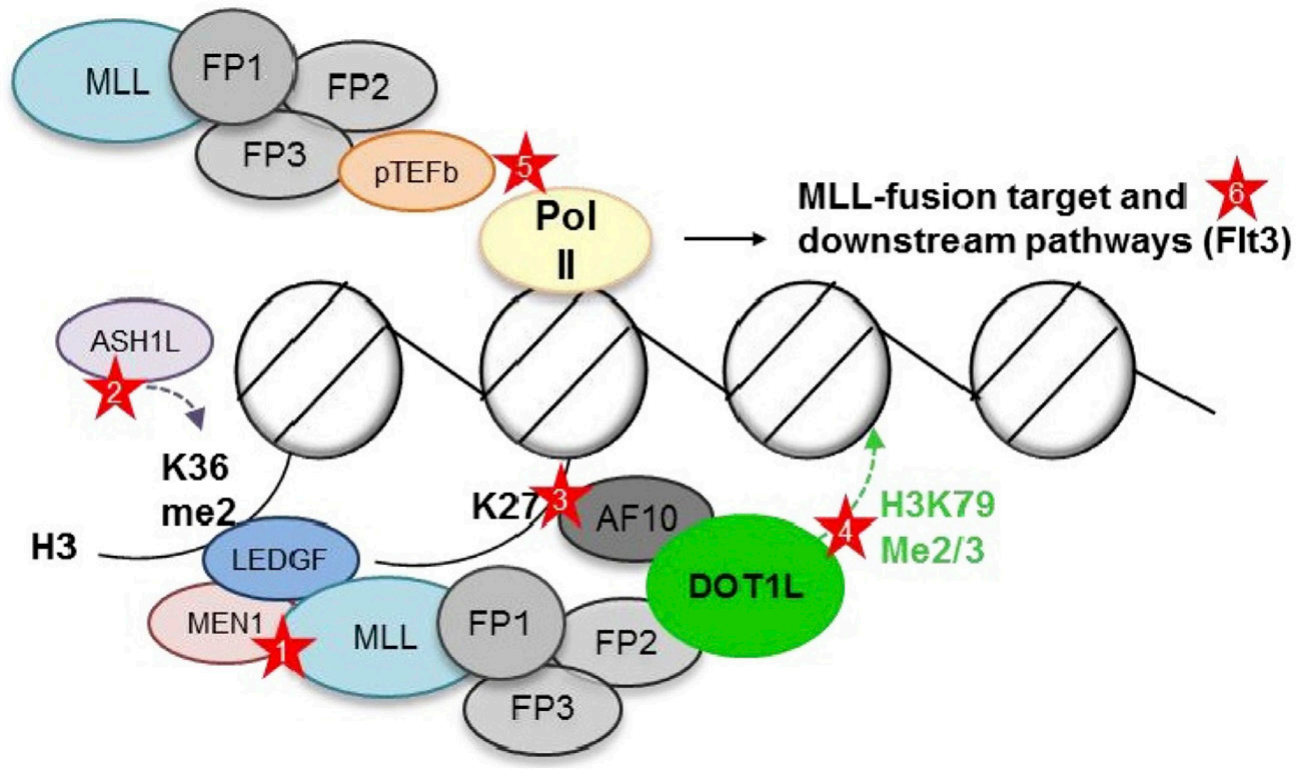
**Putative complexes between mixed-lineage leukemia (MLL) fusions and nuclear proteins involved in histone modifications and transcriptional elongation**. MEN1, Menin; FP, fusion partner. AF10, AF17, AF9, ENL, ELL, AF4, and AF5 have all been reported as MLL1 fusion partner, as well as interaction partner with each other, DOT1L, and pTEFb. However, DOT1L and pTEFb most likely do not reside within one large complex (73, 74). *Red stars depict opportunities for targeted inhibition—Protein–protein*: (1) Menin-MLL1 interaction. *Chromatin*: (2) LEDGF—H3K36me2 interaction (blocking reader domain or ASH1L). (3) AF10—unmodified H3K27 interaction (blocking reader domain or demethylases?). (4) DOT1L—placement of H3K79me2/3 (blocking methyltransferase domain). *Pol II phosphorylation*: (5) Inhibition of pTEFb. *Downstream targets*: (6) FLT3.

The cooperation of most major MLL1 fusion partners in a single elongation regulatory complex, termed “super elongation complex” (SEC), “AF4/ENL family protein complex,” or “ENL-associated protein complex”, offered an elegant explanation for the large number of different partners: translocation of any of the members of a large complex containing AF10, AF17, AF9, ENL, ELL, AF4, AF5, pTEFb, and DOT1L would cause aberrant transcriptional elongation and similar phenotypes. However, such a super complex containing an MLL fusion has remained elusive, and careful mapping of binding sites has shown that binding of several of these members is mutually exclusive, suggesting several smaller, rather than one large complex [(46–48, 73, 74); Figure 2].

Furthermore, this theory did not explain the different clinical phenotypes observed in dependence of the fusion partner (discussed below). It also did not provide an explanation for the transforming activity of MLL fusions with partners such as cytosolic coiled coil domain proteins, CBP, septins, and the MLL partial tandem duplication (PTDs). Despite a vast amount of mechanistic knowledge, *MLL* rearrangements thus still defy a simple and unifying theory of how they cause leukemia.

### The Gene Expression Program Controlled by MLL1 Fusions

Genome-wide comparisons of gene expression in *MLL-r* vs *MLL* wild-type leukemias have consistently demonstrated that this set of leukemias—irrespective of fusion partner or myeloid vs lymphoid differentiation—is distinct from all other leukemia subtypes with respect to its gene expression signature (75–77). The most frequently overexpressed genes in *MLL-r* leukemias are the later *HOX* cluster genes (particularly *HOXA7-HOXA10*) and the *HOX* cofactor *MEIS1* (78, 79). *HOX* genes encode transcription factors whose activities control developmental processes such as segmentation and hematopoiesis (31, 34, 78, 80). In these functions, they appear to have somewhat redundant roles (80, 81). In the hematopoietic system, both *HOX* genes and *MEIS1* are expressed at highest levels in the stem cells and early lineage progenitor cells, and expression levels are downregulated with differentiation (80, 81). Persistent expression of both *MEIS1*and *HOX* genes has been observed in a wide variety of leukemias (78, 82). Investigations into the dependence of *MLL-r* leukemias on upregulation of these genes have shown that *MEIS1* is necessary for leukemia growth and proliferation and that levels of expression of *MEIS1* correlate inversely with disease latency (83). The dependence of *MLL-r* leukemias for individual *Hox* genes appeared somewhat less consistent, likely due to functional redundancy among *HOX* members (81, 84, 85). However, it seems safe to say that dysregulated expression of the *HOX* developmental regulators and their cofactor *MEIS1* contributes critically to the stem cell-like characteristics of *MLL-r* leukemias and confers or maintains on these cells self-renewal properties, growth, and survival advantages that promote their oncogenic potential.

These stem cell-like properties—which may also in part depend on the developmental stage at which the leukemia arose (stem cell vs early progenitor)—have been proposed to contribute to the high level of resistance to programmed cell death frequently observed in the clinic (86–91). In addition, frequent dysregulation of prosurvival pathways such as BCL-2, which counteracts the intrinsic mitochondria-mediated apoptotic pathway, may contribute to the therapeutic difficulties many of these leukemias pose in the clinical setting (89, 92).

## Clinical Features of MLL-*r* Leukemias

### Demographics and Common Features

As the name suggests, MLL rearrangements are found in mixed-lineage leukemias [now named mixed phenotype acute leukemia (MPAL) (93)]. For the most part, however, leukemias arising from rearrangements of the *MLL* gene manifest as either acute lymphoid or acute myeloid leukemias (ALL or AML, respectively), and only a minority of MPAL actually carry *MLL* rearrangements. *MLL-r* leukemias make up approximately 10% of acute leukemias in all age groups (94). There is a bimodal distribution of affected patients, with *MLL* rearrangements most commonly found in ALL in infants less than 12 months of age and in a much broader age range of older children or adults, with AML slightly more common than ALL in this age range (94). Finally, there is a rare entity known as “therapy-related leukemia,” which typically occurs after exposure to topoisomerase II inhibitors (e.g., etoposide, doxorubicin) (95, 96).

In the case of infant leukemias, the incidence of *MLL* rearrangements is 70–80% (29, 97). Therapy-related leukemias secondary to the aforementioned chemotherapeutic agents also harbor *MLL* translocations in at least 70% of cases (98, 99). Of all patients treated with topoisomerase II inhibitors, between 2 and 12% go on to develop secondary leukemias (100). The majority of these are AML, although a smaller number of cases of ALL have also been reported (96). The latency period for this group of leukemias, in contrast to leukemias secondary to other types of carcinogens, is extremely short—as early as 6 months postexposure, and generally within 24–48 months of exposure, to topo-II inhibitors (95, 96, 98, 100). The mechanisms behind the development of *MLL-r* leukemias will be explored in the “Environmental and Genetic Risks” section.

*MLL-r* as a subgroup of acute leukemias is associated with certain phenotypic features that set it apart from other classes of leukemias. *MLL-r* acute leukemias, particularly in infants, are more likely to present with hyperleukocytosis and CNS involvement (101–103). In cases of *MLL-r* B-ALL, the blasts are typically of the pro-B phenotype and lack expression of CD10/common acute lymphoblastic leukemia antigen and frequently show coexpression of myeloid markers (104). This is also true in many cases of *MLL-r* leukemias in adults (105). *In vitro, MLL-r* blasts often have resistance to commonly used chemotherapeutic drugs such as prednisone and l-asparaginase, but typically have acute sensitivity to cytarabine (106). It has been reported that the transporter protein that imports Ara-C across the cell membrane, the human equilibrative nucleoside transporter 1, was expressed at 2.5-fold higher levels in a cohort of leukemia cells with *MLL* rearrangements than in *MLL* wild-type leukemias (107). It is possible that enhanced transport of Ara-C across cell membranes leads to preferential accumulation of the drug in *MLL-r* cells, which contributes to their specific sensitivity.

### Common MLL Fusion Partners and Lineage Plasticity

**Figure 3:**
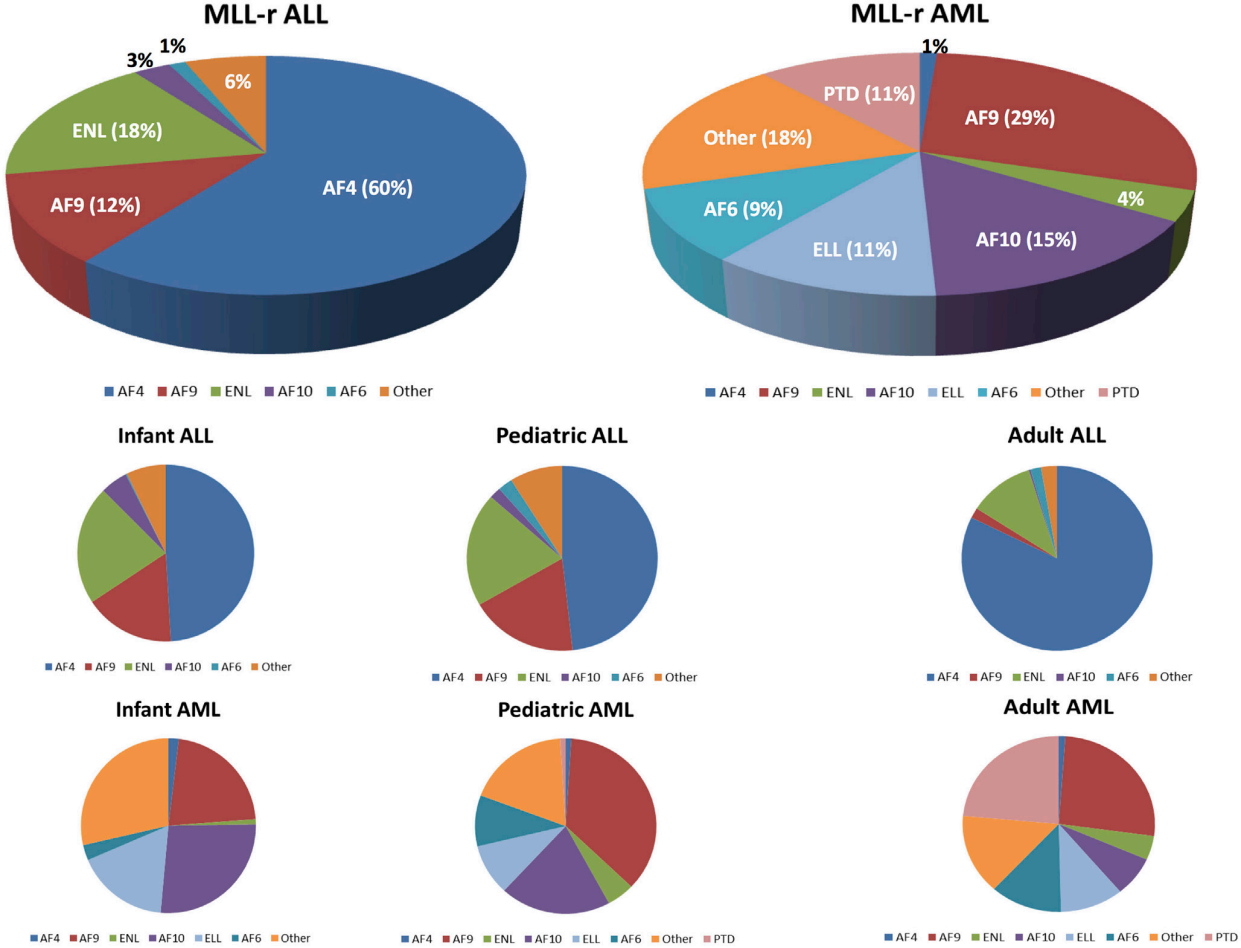
**Mixed-lineage leukemia (MLL)-rearranged leukemias involve fusions of 11q23 with 1 of more than 80 different partner genes**. Six or seven fusion partners are responsible for the majority of cases. The pie chart illustrates the relative frequencies of the different fusion partners in acute lymphoblastic leukemia (ALL) and acute myeloid leukemias (AML), respectively. Numbers are adapted from the published data by Meyer et al. (29). The bottom half of the figure shows the breakdown of the relative frequencies of MLL fusion partners based on the leukemia type (ALL vs AML) and age group.

The majority of *MLL-r* leukemias involve fusions of *MLL* with one of six common partner genes: AF4 [t(4,11)], AF9 [t(9,11)], ENL [t(11,19)(q23,p13.3)], AF10 [t(10,11)], ELL [t(11,19)(q23,p13.1)], or AF6 [t(6,11)] (29). The relative frequency of these fusions with respect to leukemia subtype and age are shown in Figure 3 [data adapted from the study by Meyer et al. (29)]. Translocations may or may not be observable on karyotype analysis, but are more reliably identified by fluorescence *in situ* hybridization (101, 104).

Clinical evidence suggests that the fusion partner of *MLL1* is a major determinant of the ultimate leukemia phenotype. In patients, *MLL-AF4* is predominantly associated with lymphoid malignancies, whereas *MLL-AF9* more often results in myeloid malignancies (29). At the same time, particularly, the lymphoid *MLL-r* leukemias retain a substantial amount of lineage infidelity and lineage plasticity. This is evident in the frequent co-expression of myeloid markers and is the phenomenon familiar to every clinician of *MLL-r* B-ALL patients who relapse with apparent AML that is cytogenetically related or even identical to the initial lymphoid disease. This phenomenon is likely to increase, as therapies directed against B-lymphoid cell surface markers enter expanded clinical use (antibodies, antibody–drug conjugates, bispecific antibodies such as blinatumomab, and CAR-T). Relapse with leukemia that has adopted a myeloid fate was recently reported for two of seven patients treated with a CD19 directed CAR-T (108) and in an infant with t(4,11) ALL treated with blinatumomab (109). This plasticity is also reflected the recurrent finding of *MLL* rearrangements in leukemias of ambiguous lineage (MPAL) (93, 110). Experimentally, Wei et al. demonstrated that the microenvironment can play a role in lineage determination. On transduction of human HSCs with a retroviral MLL-AF9 construct, transformed cells propagated in culture with cytokines that promote myeloid differentiation invariably expressed myeloid surface markers (111). Despite the association of MLL-AF9 with myeloid features, transformed cells exposed to cytokines that promote lymphoid differentiation expressed both B cell and myeloid markers. Importantly, leukemia cells of different phenotype from lymphoid or myeloid culture were found to be clonally related, suggesting that they arose from a single leukemia stem cell. Therefore, although the fusion partner affects the leukemia phenotype, environmental cues and selective pressure can also contribute.

In addition to translocations, in-frame PTD of exons 5-12 or a portion thereof can be seen in acute leukemias (112, 113). This type of *MLL* mutation was originally described in adult *de novo* AML patients with normal karyotype and has since been demonstrated in both childhood and adult ALL and AML as well as in therapy-related leukemia (112), with an overall incidence of 5–10% (113). *MLL-PTD* has also been found in a number of leukemias with extra copies of chromosome 11 (114). The presence of this abnormality is associated with early relapse of disease following initial remission (113, 114).

### Environmental and Genetic Risks

When translocated, disruption of the *MLL* gene typically occurs within the breakpoint cluster region (BCR), which spans an 8.3-kb region from exon 8 to exon 14, inclusive (115, 116). A number of sites within this portion of *MLL* are vulnerable to damage. Among them are the scaffold attachment regions (SARs), which are areas of contact between DNA and non-histone proteins of the chromatin scaffold. Two such SARs have been identified within the *MLL* coding region—one 5’ to the BCR and a stronger one within the 3’ part of the BCR (117). Cleavage sites of topoisomerase II are also found scattered throughout the *MLL* BCR, with a higher density in the SAR that overlaps the 3’ region of the BCR. Topoisomerase II is an enzyme that is essential for the relaxation of supercoiled DNA during chromatin remodeling processes (100, 116). Drugs that inhibit the enzyme, such as epipodophyllotoxins and certain alkylating agents, typically do so by forming a stable ternary complex with the enzyme and DNA. The resulting double-strand breaks are most likely to be repaired by non-homologous end joining.

Topoisomerase II inhibitors such as etoposide are known to be associated with development of **MLL**s in therapy-related cases, and the breakpoints within the *MLL* gene are frequently adjacent to known cleavage sites of the enzyme (116, 118). Potential *MLL* cleavage by unknown apoptosis-activated proteases that seem to act independently of topoisomerase II has also been reported in response to stimuli such as ionizing radiation (119). Interestingly, the majority of *MLL* breakpoints identified in infant leukemias lie toward the 3’ end of the BCR, similar to those seen in leukemias secondary to treatment with topoisomerase II inhibitors (115–117). This finding suggests a possible common mechanism for these two groups of leukemias. Along those lines, investigations into prenatal exposures of infants with *MLL-r* leukemia have suggested that bioflavonoids found in foods and herbal remedies such as dipyrone (“Mexican aspirin”); senna in herbal teas; quercetin, a bioflavonoid found in onions, red wine, and other foods; and genistein found in soybean products could act as inhibitors of topoisomerase II (100, 120, 121) and promote rearrangement of the *MLL* locus in a variety of cells, including CD34+ hematopoietic progenitor cells (120, 122). Therefore, it seems possible that, at least in some instances, *in utero* exposure to environmentally occurring topoisomerase II poisons could contribute to the development of *MLL-*rearrangements. However, most of these agents are very common, and infant leukemia is a very rare disease. This discrepancy has not well resolved and suggests additional stochastic or genetic mechanisms.

It is also important to note that, in very young patients, there is substantial evidence that the gene rearrangement usually, if not always, occurs *in utero*. Polymerase chain reaction (PCR) testing of neonatal blood spots has demonstrated the presence of translocations involving the *MLL* gene even in babies whose disease was diagnosed months later (123). Twin studies offer further support for the prenatal origin of these leukemias. The concordance rate for infant leukemia in identical twins is predicted to be close to 100%, and siblings typically have identical *MLL* breakpoints (123, 124). These observations suggest a transplacental transfer of leukemia cells from one twin to the other. Despite a rate of twin–twin transfusions of 8% in dichorionic twins, the rate of transplacental seeding of leukemia is much lower in this situation. Both immune-mediated and genetic mechanisms may be responsible for this discrepancy. In fact, there is emerging evidence that genetic risk factors contribute to *MLL-r* leukemogenesis. In a remarkable GWAS study, a rare polymorphism in *MLL3* was present in 100% of infants with *MLL-r* leukemia (125). The mechanistic implications of this variant are currently being explored.

## Treatments and Outcomes for MLL-*r* Leukemias

### Principles and Outcomes of Multiagent Chemotherapy

Historically, the 5-year event-free survival (EFS) for infant ALL has ranged from 20 to 40% for those with *MLL* rearrangements, vs 60% or higher for those with wild-type *MLL* (102, 104). The most recent studies for whom published long-term survival data exist have indicated only modest improvement in these numbers [4-year EFS of 40–50% and overall survival (OS) of 50–55%] (102, 126, 127). Most patients (~80–90%) will go into remission initially, but relapse rates of 50–60% are reported, the most common site of relapse being the bone marrow (128). Intensification of chemotherapy may reduce the risks of relapse, but comes at the cost of significant therapy-related morbidity and mortality, mostly of infectious etiology (104). In contrast, the cases of *MLL-r* AML in infants do not generally have worse outcomes than their non-*MLL-r* AML counterparts (129). Pediatric patients greater than 1 year of age with *MLL-r* ALL are better than infants, although not as well as their non-*MLL-r* counterparts. Most recent data estimate a 5-year EFS of ~60% (129) compared to ~92% in pediatric ALL overall (130). In a European study of 85 adult ALL patients with t(4,11) rearrangements (105), 5-year EFS and OS were 34 and 35%, respectively, which is slightly diminished compared to ~40–45% long-term survival in adult ALL overall (131). Again, most *MLL-r* patients achieve an initial remission (>90%), but many patients ultimately relapse.

The relationship between MLL rearrangements and outcome in AML is less straightforward than in ALL. The most common MLL fusion in AML, MLL-AF9, has been reported to be associated with an intermediate to good prognosis (132, 133). In contrast when analyzing a large cohort of pediatric *de novo* AML with a variety of different *MLL* rearrangements, 5-year EFS and OS were poorer [44% EFS and 56% OS (133)] when compared to pediatric AML in general [55% EFS and 70% OS (134)], with substantial differences depending on the fusion partner.

Clinical features that have been shown to be predictive of outcome in infant *MLL-r* ALL include age at diagnosis, total white blood cell count at diagnosis, presence or absence of CD10 on blast cells, and initial response to steroid therapy (97, 104, 126, 128, 129). Age cutoff predictive of poorest outcomes varies based on the study (<90 days vs <6 months). WBC count >300K, lack of CD10 expression, and poor response to prednisone (defined as >1,000 blast cells per microliter in the peripheral blood) all confer particularly dismal outcomes as well (97, 104, 126, 128, 129). In adult *MLL-r* ALL, older age (>25 years) was the only independent factor associated with decreased survival [<35 vs 71% (105)].

Historically, it was thought that t(4,11) fusions in ALL were associated with poorer survival compared to other translocations (128). However, despite the association of t(4,11) and t(11,19) fusions with younger age groups, more recent trials have failed to find any significant association between relapse or survival in *MLL-r* ALL and any particular fusion partner (97, 104, 126, 129). This may be related to the fact that despite a myriad of fusion partners being reported, only a few dominate the clinical experience. Furthermore, *MLL-r* leukemias are typically not treated on a unified protocol, but managed largely based on phenotype (AML vs ALL) and age (infant leukemia). Most current clinical risk stratifications do not take the fusion partner into account. However, several studies investigating the relationship between fusion partner and outcome have suggested that there is a correlation. A meta-analysis of the association between fusion partner and outcome in 756 children with *MLL-r* AML from 11 study groups operating in 15 countries suggested massively divergent OS: while 24 children with the t(1,11)(q21,q23) translocation (*MLL-AF1q*) had an OS of 100% (event free survival, EFS 92%), EFS and OS were 11 and 22%, respectively, for patients with the t(6,11)(q27,q23) translocation (*MLL-AF6*) (133). This study did not confirm the possible “good risk” feature of the common *MLL-AF9* translocation that was previously reported (132). The dismal outcome for *MLL-AF6* mutant disease had previously been reported in adults (135). Also, children older than 1 year with *MLL-AF4* (and interestingly, *MLL-AF9*) mutant B-ALL were reported to have a worse outcome than children with other *MLL* translocation partners (129). In an even more fascinating twist, *MLL-AF9* might be predictive of a good OS when it occurs in FAB M5-AML as opposed to other FAB subgroup AML or ALL PIMD (133). Whether this reflects statistical outliers in an increasingly smaller and more finely sliced “pie,” genetic/pharmacogenomics differences, or underlying biology (possibly reflecting cell of origin) is unclear.

One interesting correlation is that both infant ALL and therapy-related leukemias, which have overall the worst outcomes of *MLL-r* leukemias, are associated with breakpoints in intron 11 rather than intron 9 or 10 (115). Emerenciano et al. recently demonstrated that the presence of *MLL* breakpoint in intron 11 was also an independent predictor of poor survival in a cohort of 30 *MLL-r* pediatric leukemia patients (136). Fragmentation of the *MLL* gene at intron 11 is predicted to generate an MLL *C*-terminal truncated protein whose PHD fingers are misfolded, eliminating the ability to associate with its repressive complex (137). The authors of this study theorize that, in these cases, unbalanced activation functions of the resultant MLL fusion protein lead to more aggressive leukemia phenotypes. Whether this can be mechanistically proven in future experiments remains to be seen.

### The Role of Hematopoietic Stem Cell Transplant (HSCT) in the Treatment of MLL-*r* Leukemia

The role of HSCT in the treatment of *MLL-r* leukemias continues to be a matter of intense debate, with several studies and meta-analyses suggesting that HSCT does not improve survival in *MLL-r* leukemias at any age group or lineage, with the exception of therapy-associated AML (126, 129, 133, 138–141). The combined analysis of the North American CCG 1953 and POG 9407 infant ALL trials concluded that HSCT failed to show any benefit (142). Initial chemotherapy was identical on the two protocols. In the later phases, chemotherapy was very similar, with the main difference being methotrexate dosing. Patients on the CCG also received maintenance therapy, while patients on the POG trial did not. On the CCG trial, HSCT in CR1 was the preferred mode of treatment if a suitable donor could be identified, whereas on the POG trial, this was left to the judgment of the investigator. The recommended conditioning consisted of Ara-C/Cy/TBI, although only about half of the patients received this conditioning. Transplant-related mortality, particularly in children receiving TBI, was high. This study included 132 infants with *MLL* rearrangement, although after adjustment for time to transplant, only 100 children were evaluable. Fifty-three underwent HSCT, 47 did not. Five-year EFS for children who were alive at the time of transplant was similar between the HSCT and chemotherapy groups (48.85 vs 48.7%), prompting the authors to conclude that HSCT did not improve survival in *MLL-r* infant ALL. In addition, there were no differences in subgroups based on WBC, age, or CD10 expression. However, the comparatively smaller number of patients with high-risk features and variability in transplant regimen made the subgroup analysis for this patient population difficult.

No benefit for HSCT for infants with *MLL-r* leukemia was also shown by two retrospective analyses (129, 143) and in a report of children treated in Europe (144). In contrast, the analysis of a larger cohort of 297 infants with *MLL* rearrangement treated on Interfant99 identified a group of patients less than 6 months of age with either a WBC of >300,000, prednisone poor response, or high end consolidation MRD that had an extremely poor survival with chemotherapy only (97, 145, 146). On Interfant99, high-risk patients did benefit from HSCT: the survival for children <6 months with either a WBC > 300,00 or PPR who were alive at the time of HSCT was only 22.2% when treated with chemotherapy and 59% on the HSCT arm (97). The number of patients who received HSCT was small, but given the dismal outcome of this subgroup, a more aggressive approach seems justified. The outcome of a similar group of infants on other trials such as CCG 1953 and POG 9407 is not known, since the number of patients was smaller, prednisone response and MRD were not assessed or reported, and WBC criteria for subgroup analysis were different from the Interfant99 study (97, 104).

In summary, although numbers are small, HSCT is likely beneficial for a defined subgroup of high-risk infants, particularly if conditioning regimen and donor choice allow for a low transplant-related mortality. There are no data supporting HSCT in CR1 in older children with *MLL-r* ALL or *de novo* AML. As discussed earlier, the study by Balgobind et al. suggests that defined fusion partners may be associated with a particularly poor prognosis (133); however, currently, this is not used for treatment stratification in either ALL or AML. In contrast to *de novo* leukemia, outcomes for therapy-related AML are substantially worse, and HSCT in first CR is standard of care (141). In addition to tAML, treatment-related *MLL-r* ALL is occasionally seen. Although outcomes for tAML are inferior to *de novo* AML, no such data exist for *MLL-r* tALL vs *de novo* ALL, and chemotherapy only may be the treatment of choice, particularly for patients who show a good response to therapy (as measured by MRD).

### Role of FLT-3 in MLL-*r* Leukemias

One of the most significantly upregulated genes in the transcriptional profile of *MLL-r* leukemias is Fms-like receptor tyrosine kinase-3 (FLT-3) (75). This gene encodes a class III receptor tyrosine kinase, which is closely related to KIT, FMS, and platelet-derived growth factor receptor (147). Under physiologic conditions, binding of the FLT-3 ligand leads to dimerization and phosphorylation of the receptor, which activates downstream signaling pathways such as PI3K/Akt, Ras/MAPK, and Stat5 (147). Activating mutations in FLT-3 have been described in a variety of hematologic malignancies, but have primarily been characterized in pediatric and adult AML (147, 148). The two types of activating mutations commonly seen in these contexts are internal tandem duplications in the juxtamembrane domain of FLT-3 [so-called FLT-3 internal tandem duplications (ITD) mutations] and point mutations within the tyrosine kinase domain (TKD) that confer constitutive activity to the enzyme (147). The presence of a FLT-3 ITD mutation confers an extremely poor prognosis (148).

FLT-3 gene upregulation in *MLL-r* leukemias correlates to overexpression of the FLT-3 protein in these ALL samples compared to non-*MLL-r* leukemias, which has been demonstrated by several groups (149–152). On average, *MLL-r* infant ALL (which is the most thoroughly studied subtype of *MLL-r* leukemias) expresses 37-fold higher FLT-3 protein compared to normal bone marrow and 2- and 16-fold higher expression of FLT-3 compared to non-*MLL-r* ALL in children less than 1 year of age and older children, respectively (150, 152). By contrast, the data have been less consistent with respect to activating mutations of FLT-3, with most of the recent studies suggesting that they are rare (149–151, 153). FLT-3 ITD have not been demonstrated in recent cohorts of *MLL-r* ALL patients, and FLT-3 TKD mutations have an approximate incidence of only 3–18% in *MLL-r* ALL (149–151). Most recently, Andersson et al. investigated a cohort of 85 infant and pediatric patients with ALL, of which 67 had *MLL* rearrangements. Of these, only four patients had FLT-3 mutations, two of which were TKD mutations and two of which were present only in a minor clone (154). This finding is also consistent with the general finding by this group that *MLL-r* leukemias, in particular those arising in infants, have one of the lowest frequencies of somatic non-silent mutations of any other type of cancer (mean 1.3 per major clone). Nevertheless, *in vitro* studies have demonstrated that high levels of FLT-3 expression, even in the absence of these activating mutations, are associated with phosphorylation and activation of the protein (149, 150).

Mouse models of *MLL-r* leukemias have suggested cooperation between the MLL fusion oncoprotein and FLT-3 in the progression to the leukemia phenotype (155). In addition, a retrospective study showed correlation of high levels of FLT-3 expression with poor outcomes in 32 *MLLr* infants treated on Interfant99 (36 vs 71% 1-year EFS in high vs low FLT-3 expressing leukemias) (156). A later study by Chillon et al. confirmed these findings—of 17 patients with *MLL-AF4* B-ALL, none of those with high FLT-3 expression were alive at 1 year, compared to 71% of patients with low FLT-3 expression (152). FLT-3 expression levels were not predictive of outcomes in patients with non-*MLL-r* ALL. These findings suggested that targeting of FLT-3 in *MLL-r* patients might be a beneficial therapeutic approach.

*In vitro* cytotoxicity experiments with *MLL-r* ALL patient samples demonstrated *in vitro* sensitivity to the FLT-3 kinase inhibitors, with response correlating with the amount of FLT-3 overexpression [PKC412 (150) and CEP-701/lestaurtinib (151)). Furthermore, synergy studies between CEP-701 and standard chemotherapeutic agents (e.g., etoposide, daunomycin) suggested that timing is critical—administration of CEP-701 after cytotoxic agents yielded synergistic cytotoxicity, whereas administration of CEP-701 before cytotoxic chemotherapy was antagonistic (157). These studies laid the groundwork for the design of clinical trials to test the efficacy of FLT-3 inhibitors.

Several clinical trials involving FLT-3 inhibitors have been conducted in both adult and pediatric leukemias. Of primary relevance to *MLL-r* leukemias is the Therapeutic Advances in Childhood Leukemia & Lymphoma (TACL) study, whose results have just been published (158). This study was a phase 1 trial evaluating the safety of quizartinib (AC220) in combination with high-intensity chemotherapy for relapsed childhood leukemia. Quizartinib is a second-generation kinase inhibitor designed to be potently active against FLT-3 and is more selective than first-generation inhibitors such as lestaurtinib (159, 160). Twenty-two patients were enrolled, of which 18 had relapsed AML (9 with FLT-3 mutations) and 4 had relapsed *MLL-r* ALL (3 infants and 1 teenager). Patients received combination chemotherapy with cytarabine and etoposide (days 1–5) followed by quizartinib (days 7–28) for 1–2 cycles. In all cases, target-specific activity of quizartinib was demonstrated with near-maximal (>95%) suppression of FLT-3 phosphorylation in plasma inhibitory assays (PIAs). Dose-limiting toxicities attributable to the targeted agent involved primarily GI toxicities such as elevated lipase or transaminases or nausea/vomiting/diarrhea. Of the 17 evaluable patients for response, better response correlated with the presence of FLT-3 ITD mutations in the AML patients. Three of the four *MLL-r* ALL patients could be evaluated for disease response—one had stable disease and two had progressive disease. The study was not powered to make conclusions about statistically significant impacts on OS.

Another trial specific to infant leukemia, the Children’s Oncology Group (COG) trial AALL0631, has been recently closed, and data analysis is ongoing. This trial was a randomized, phase III trial of the FLT-3 inhibitor lestaurtinib in combination with intensive cytotoxic chemotherapy for newly diagnosed infants with *MLL-r* ALL. Although the final results of the trial have not yet been published, the results from the TACL trial raise concern that FLT3 inhibition may not be the breakthrough that is so desperately needed for these patients. However, final results on the outcomes and the depth of FLT-3 inhibition achieved in AALL0631 regimen is not (yet) known, thus failure to achieve sufficient target inhibition remains a possible explanation for the lack of efficacy. It is also critical to keep in mind that in both AALL0631 and the TACL study, the assays used to determine the degree of FLT3 inhibition measures the inhibitory effect of patient serum on BAF3 cells that are Flt3 dependent (PIA). The threshold at which this very sensitive indicator cell line responds may be different from responses in patients’ leukemia cells. It may be necessary to determine on-target activity in actual patient cells and correlate that with response to get a better sense for whether FLT3 inhibition is of therapeutic value in *MLL-r* leukemia.

### Proteasome Inhibitors

Proteasome inhibitors are increasingly being integrated into therapeutic regimens for a variety of malignancies. The rationale behind their use has traditionally been that cancer cells, due to increased cell turnover, are more dependent on the proteasome machinery for protein recycling than are normal cells. These drugs have yielded mixed results when used alone or in combination with cytotoxic chemotherapy for a variety of malignancies and are associated with significant toxicities, particularly neurotoxicity [reviewed in Ref. (161)].

Accumulating data now suggest that proteasome inhibitors may be promising agents to supplement the treatment of *MLL-r* leukemias. Liu et al. noted that the expression levels of MLL fusion proteins was not excessive in leukemic cells and hypothesized that tight regulation of fusion protein expression might be achieved through the proteasome machinery. Indeed, they demonstrated that proteasome inhibitor treatment increased the protein levels of both wild-type MLL and, to a greater extent, MLL fusion proteins (162). Stabilization of MLL fusions activated transcription of CDKN1B, which encodes p27, *via* PAX5. Of note, PAX5 is selectively expressed in pro-B cells (163). Consistent with this model, proteasome inhibitor treatment was associated with a dose-dependent decrease in cell viability in lymphoid, but not myeloid, *MLL-r* leukemia cell lines. A cohort of five adult patients with *MLL-r* leukemias were then treated compassionately with single agent bortezomib. Three patients (two with pro-B phenotype and one with biphenotypic leukemia) had transient hematologic responses to the drug, one for over a year. Two patients with myeloid phenotype had no response to proteasome inhibitor therapy.

Bortezomib was also identified through high-throughput drug screens as an active agent against models of infant *MLL-r* leukemias (164, 165). Mechanistically, Koss et al. found that bortezomib treatment led to decreased histone 2B ubiquitination (H2Bub). H2Bub is required for the methylation of H3K79 mono- to di- and trimethylation (166), and knockdown of the H2B ubiquitin ligase RNF20 led to decrease in H3K79me2 mark at MLL target gene sites and *in vitro* and *in vivo* compromise of leukemia cell viability (167). Both the study by Liu et al. (162) and Wang et al. (167) also explored the effect of bortezomib in NF-kB signaling. Intact NF-kB has been reported to be required for MLL-fusion-mediated leukemogenesis in a murine model (168, 169), and bortezomib has been implicated in negatively regulating NF-kB *via* the accumulation of IkBa in the absence of proteasomal degradation (170). However, both Liu et al. (162) and Koss et al. (164) found no evidence of NF-kB modulation, suggesting that this pathway is not mechanistically involved in therapeutic effect. Taken together, proteasome inhibitors may be useful as adjunctive therapy for *MLL-r* leukemias, and bortezomib in combination with standard chemotherapy and HDAC inhibition is currently being evaluated in clinical trials for *MLL-r* ALL (NCT02419755, see next section).

### HDAC Inhibitors

Similar to proteasome inhibitors, HDAC inhibitors (HDACi) have been reported by multiple groups to be active in *MLL-r* leukemias, with effects being attributed to diverging mechanisms (165, 171–174). HDACs are a large family of proteins named for the ability of the founding member, HDAC1, to deacetylate histones. However, many members of the family are cytosolic proteins named HDAC due to structural homology, but without possessing histone deacetylase function. Several HDACs have been reported to be overexpressed in pediatric ALL; however, there is no agreement as to which members are specifically deregulated (175–177).

The earliest functional relevance of HDACs was suggested by a study investigating the activity of valproic acid in *MLL-r* leukemia. Valproic acid induced growth inhibition and cell-cycle arrest in *MLL-r* leukemia cell lines and primary samples. The authors proposed upregulation of p21 as a mechanism (171). Stumpel et al. showed that romidepsin (FK288) and vorinostat had activity in 2 t(4,11) B-ALL cell lines and 15 infant B-ALL patient samples (172). Good, although not as profound, sensitivity to HDAC inhibition was also found for non-*MLL* rearranged B-ALL. They also demonstrated decrease in expression of MLL-AF4 at both the transcript and protein levels, raising the possibility that effect of these drugs is primarily due to downregulation of the MLL fusion itself.

In contrast, Stubbs et al. found that several HDACis were broadly active against a variety of different cytogenetic subtypes of B-ALL, including (but not exclusively) *MLL-r* leukemias (174). Genetic knockdown as well as class-specific inhibitors suggested that HDAC1 and 2 are the critical HDACs in B-ALL, with knockdown or inhibition resulting in direct cytotoxicity and DNA damage. DNA damage as a result of HDAC inhibition has been suggested to underlie the frequently observed synergy with chemotherapy. Stubbs et al. proposed that the particular sensitivity of B-ALL to HDAC inhibition could also relate to the role of HDAC1 and HDAC2 in early B-cell development (178). On the other hand, two studies suggested that inhibition of HDAC3 is critically responsible for the activity of HDACis in B-ALL (179, 180).

Bhatla et al. published sensitivity of the t(4,11) B-ALL cell line RS4,11 to the HDACi vorinostat, and vorinostat was shown to be synergistic with standard chemotherapeutic agents such as prednisolone and cytarabine (173). The authors of this study also specifically investigated relapsed B-ALL (irrespective of karyotype) and proposed the reversal of the “relapse gene signature” as a mechanism. Finally, an interesting mechanism was proposed by Ahmad et al., who proposed that HDACis reactivate wild-type MLL to counteract the transcriptional functions of MLL-AF4 or other fusions (181). However, the role of wild-type MLL in *MLL-r* leukemia is controversial as discussed above, and the experiments by Ahmad et al. were performed in HeLa cells, with unclear implications for the context of leukemia.

From a clinical standpoint, a 2011 case study reported a sustained complete cytogenetic response to single-agent panobinostat in an elderly man with therapy-related *MLL-r* leukemia (182). St. Jude Children’s Research Hospital has an ongoing phase II clinical trial combining a proteasome inhibitor (bortezomib) and an HDACi (vorinostat) in combination with cytotoxic chemotherapy for pediatric patients with relapsed or refractory *MLL-r* leukemias (NCT02419755). Chemotherapy backbone varies depending on the leukemia phenotype (ALL vs AML), and all drugs are intended as a bridge to transplant. A report of six “pilot” patients with relapsed/refractory *MLL-r* leukemia was presented at the 2014 American Society of Hematology Annual Meeting. The overall response rate of this cohort to chemotherapy in combination with bortezomib and vorinostat was 83%: four patients had complete response, one patient had partial response, and one patient had stable disease (164). Whether this regimen can achieve durable responses without excess toxicity in these patients remains an ongoing question, but initial results are certainly promising.

### Hypomethylating Agents

Two separate groups have investigated the methylation status of *MLL-r* leukemias compared to *MLL-*wild-type leukemias and normal controls (183, 184). Global promoter hypermethylation was seen in the *MLL-r* leukemias relative to both non-*MLL-r* leukemias and normal samples, leading to downregulation or silencing of a subset of tumor suppressor genes. Retrospective analysis demonstrated a statistically significant correlation between degree of methylation and risk of relapse (183). Furthermore, hypomethylating agents zebularine and decitabine showed preferential cytotoxicity to *MLL-r* cells compared to other leukemic cells. Both decitabine and another hypomethylating agent, 5-azacitidine, are FDA approved for treatment of myelodysplastic syndromes and AML in adult patients, particularly those with co-morbidities limiting other therapeutic options [reviewed in Ref. (185)]. 5-azacitidine, but not decitabine, usage was associated with increased OS in these patients. These data form the basis for a clinical trial run by the National Cancer Institute (NCT02828358), and for a new COG therapy trial (AALL15P1), both for infant *MLL-r* ALL, which test the tolerability of 5-azacitidine in combination with standard cytotoxic chemotherapy.

Similarly to decitabine and 5-azacitidine, other nucleoside analogs may also target the methylation status of *MLL-r* leukemias. Clofarabine, an powerful cytotoxic adenosine analog, is thought to also block DNA methylation through depletion of S-adenosyl methionine, which donates methyl groups to DNA methyltransferase enzymes (186), as has been demonstrated for the related molecule cladribine (187). In a recent study by Stumpel et al., low doses of clofarabine were cytotoxic to *MLL-r* leukemias *in vitro*, and clofarabine was synergistically active with cytarabine against these cells (188). A variety of clinical trials, none specific to *MLL-r* leukemias, currently incorporate clofarabine into study therapy.

### Immunotherapy

One of the most exciting new therapeutic approaches in B-ALL has been the development of immunotherapies, particularly the use of bispecific antibodies (blinatumomab) and engineered T-cells (CAR-T) in B-ALL. Most clinical trials using CAR-T cells in B-ALL to date have allowed children >1 year and adults with *MLL* rearrangements, but have excluded infants, mostly due to difficulties around efficient collection and expansion of autologous T-cells. Infants were included in the early clinical trials with blinatumomab (189), and despite theoretical concerns about the immaturity of T-cell responses very early in life, some encouraging responses were seen [Lia Gore, personal communication (109)]. However, *MLL-r* B-ALL may have “built-in” mechanisms to evade immune recognition and/or destruction through their lineage plasticity. As mentioned earlier, relapse with leukemia that has adopted a myeloid fate has been observed in two out of seven patients treated with a CD19-directed CAR-T (108) and in an infant with t(4;11) ALL treated with blinatumomab (109). It is not yet clear whether this will remain a rare occurrence or emerge into a common mechanism of relapse and resistance.

## MLL Specific Pathways and Targeted Inhibitors in Early Clinical Trials

### Role of RAS Pathway Mutations in MLL-*r* Leukemias

Mutations in RAS pathway members have been frequently described in *MLL-r* leukemias and are perhaps more prevalent than mutations in FLT-3. Prelle et al. evaluated the incidence of secondary mutations in a cohort of 144 pediatric and adult patients with *MLL-r* leukemias, of which 100 individuals had t(4;11) mutations and the remaining 44 patients had a variety of other fusions. NRAS or KRAS mutations were present in 16 patients (11.1%) (153). In an independent cohort of 109 infant ALL patients screened for NRAS, KRAS, or BRAF, 15 patients (13.8%) had mutations in either NRAS or KRAS (190). This group also reported a significant decrease in OS was specifically seen in patients with RAS mutations in the t(4;11) cohort, but not in the overall study group. Additional studies have cited frequency of RAS pathway mutations in MLL-r leukemia patients ranging from 22 to 45% (191–194).

Recently genome-wide analysis of infant *MLL-r* and *MLL*-wild-type ALLs, in addition to pediatric *MLL-r* ALL in older age groups, was performed as a part of the Pediatric Cancer Genome Project (154). This study confirmed that although *MLL-r* leukemias in general carry a paucity of additional mutations, the most commonly seen mutations involve the RAS pathway. However, the variant allele frequencies (VAF) for these mutations in the majority of the infant cases were <30%, indicating that the individual mutations were present in minor clones within the leukemia population. This was also noted for the NRAS and KRAS mutations described in the study by Driessen et al. (190). Furthermore, of five RAS pathway-mutated patients with matched diagnosis and relapse samples, the mutations were lost in two cases and the VAF decreased in one case, suggesting a gradual depletion of the RAS-mutated subclone (154). Again, this mirrors previous studies by Prelle et al. and Emerenciano et al., both of whom documented loss of RAS mutations in two of three and in five of 18 relapse samples evaluated, respectively (153, 192). Furthermore, Emerenciano et al. documented the presence of RAS mutations in DNA samples from newborn blood spots for two patients in their cohort, one with higher allele frequency than at diagnosis of leukemia. This finding suggests the possibility that RAS pathway mutations provide a proliferative advantage during onset of leukemogenesis, but are not necessary for leukemia maintenance in the context of *MLL* rearrangements.

With the advent of multiple inhibitors of the RAS pathway that are either FDA approved or in clinical trials, the question whether RAS pathway activation plays a role in *MLL-r* leukemia receives new urgency. It is also possible, similar to FLT3, that activation of the pathway can occur in the absence of mutations. Kampen et al., using peptide arrays of normal bone marrow and leukemia cells, demonstrated increased phosphorylation of MAPK pathway proteins in *MLL-r* AML samples compared to either normal bone marrow or non-*MLL-r* AML’s (195). MEK inhibitors have shown selective activity against *MLL-r* leukemia cell lines and primary samples *in vitro* in several studies, although in almost every case those cells with RAS mutations were more sensitive to these drugs than were cells without RAS mutations (193–195). The possible exception to this rule lies in leukemia cells harboring t(6;11), leading to an MLL-AF6 fusion. Manara et al. have shown that the normally cytoplasmic protein AF6 is instead localized to the nucleus in the presence of MLL-AF6, which is associated with increased RAS pathway activity. The AF6 protein has RAS-association domains, and genetic silencing of MLL-AF6 leads to decreased RAS activity and decreased phosphorylation of ERK (196). Furthermore, chemical inhibition of RAS signaling by either PD98059 (MEK inhibitor) or tipifarnib (farnesyltransferase inhibitor) was selectively toxic to t(6;11) leukemia cells. Therefore, although RAS inhibition may not be of benefit in the majority of *MLL-r* leukemias, where mutations are subclonal and not likely to impact the survival of the leukemia, it may be of benefit in the context of leukemias with MLL-AF6 fusions, which are notorious for their particularly poor outcomes.

### Dot1L Inhibitors

The histone 3 lysine 79 methyltransferase Dot1L has been shown to be necessary for MLL fusion-mediated transformation in a variety of experimental models (45, 70, 197–200), and increased levels of H3K79 dimethylation have been demonstrated at MLL fusion target gene loci [(70, 198, 201); see also Figure 2]. DOT1L contributes to the maintenance of the MLL leukemic gene expression program at least in part by antagonizing Sirtuin-1-mediated repressive epigenetic modifications to H3K9 (202).

A small molecule inhibitor of Dot1L (EPZ-5676 or pinometostat) has been developed by Epizyme, Inc. and is being studied in early clinical trials in both adult and pediatric patients with *MLL-r* leukemias (NCT01684150 and NCT02141828). Preclinical studies demonstrated target specificity and downregulation of MLL target genes upon treatment of *MLL-r* cell lines with EPZ-5676 (203). EPZ-5676 also demonstrated synergy with other chemotherapeutic agents known to target *MLL-r* leukemias, regardless of the order of administration (204). Continuous intravenous infusion of the compound caused tumor regression and prolonged survival in mice and rat xenograft models of *MLL-r* leukemias (203). Unfortunately, the lack of oral bioavailability and short half-life of the drug currently mandate the continuous IV infusion, resulting in efforts to develop alternative Dot1L inhibitors, which maintain specificity and efficacy but are easier to administer (205). Data from the phase I/II clinical trials are forthcoming, but it will be crucial to correlate mechanistic effect (i.e., reduction of H3K79 methylation) with outcomes in these patients. Preliminary data published in abstract form suggest sustained single-agent efficacy in two patients, but also a substantial number of patients with only transient or no response, and who did not achieve profound depletion of H3K79 methylation at MLL-fusion target loci at the dose used (206, 207).

### Bromodomain Inhibitors

The bromodomain and extra terminal (BET) family of proteins, which includes BRD2, BRD3, and BRD4, are a family of chromatin adaptor proteins that recognize and bind to acetyl-lysine residues. A global proteomic screen identified interaction of these proteins with components of the SEC, many of whom are MLL fusion partners (208, 209). Simultaneously, an shRNA screen identified the BET protein BRD4 as a therapeutic target in MLL-AF9,Nras^G12D^ murine AML model (210). Inhibitors of BRD4 had efficacy in MLL-AF9,Nras^G12D^ model and on a variety of leukemia cell lines, with *MLL-r* leukemias preferentially affected (209, 210). Efficacy of BRD4 inhibition was confirmed in primary *MLL-r* patient samples *in vitro* (165, 209).

In addition to the bromodomain of BET family proteins, the bromodomain of CBP/p300 bromodomain has emerged as a potential therapeutic target for leukemia, including *MLL-r* leukemia. A small molecule inhibitor of the CBP/p300 bromodomain led to decreased colony formation and promoted differentiation in *MLL-CBP* and *MLL-AF9* leukemia models as well as primary *MLL-r* patient cells (211). This latter inhibitor was found to have synergistic inhibition of *MLL-r* cells when combined with the bromodomain inhibitor JQ1 or doxorubicin.

One recent study suggested a sequential recruitment of DOT1L and BRD4 to a subset of genes located adjacent to super enhancers (212). Dimethylation of H3K79 by DOT1L allowed binding of histone acetyltransferases including EP500 and CREBBP to these regions, which leads to acetylation of H4K5 and subsequent binding of BRD4 and the SEC. Accordingly, inhibition of DOT1L led to dramatically decreased binding of BRD4 to chromatin, and the combination of a bromodomain inhibitor and a DOT1L inhibitor was synergistically active against *MLL-r* leukemias both *in vitro* and *in vivo*. In contrast, regulation of distinct programs by DOT1L and BRD4 were reported by Garcia-Cuellar et al. (213). DOT1L-dependent loci were characterized by promoter-centered binding of MLL-ENL, while BRD4-dependent loci exhibited fusion binding beyond the termination site. Despite the discrepancies in proposed molecular mechanisms, the combination of DOT1L and BRD4 inhibition may be promising to explore further.

### Lysine-Specific Demethylase-1 (LSD1) Inhibitors

Lysine-specific demethylase-1, also known as KDM1A, has been shown to be important for maintenance of MLL target gene expression (214) and was identified in an RNAi screen as a gene whose repression inhibited growth of MLL-AF9,Nras^G12D^ murine cells (215). It has enzymatic specificity for lysines 4 and 9 on histone 3. Pharmacologic inhibition of LSD1 with tranylcypromine (TCP) was shown to result in a decreased expression of MLL target genes; it also impaired colony-forming potential and leukemic engraftment in immunodeficient mice (214). However, there were significant toxicities to the mice from TCP and related inhibitors, particularly related to thrombocytopenia and anemia. Using newer generation small-molecule inhibitors of LSD1, these results were confirmed *in vitro* and *in vivo* without any significant toxicities to mice (216). Furthermore, *in vitro* synergy was demonstrated when LSD1 inhibitors were combined with the DOT1L inhibitor SYC-522. However, Shi et al. were only able to demonstrate a detrimental effect of LSD1 inhibition *in vitro*, whereas no disadvantage of LSD1 inhibition could be shown in competitive engraftment experiments in mice (215). Pharmacologic inhibitors of LSD1 are in early clinical trials in adult AML/MDS (GSK GSK2879552 single agent, NCT02177812, Tranylcypromine + ATRA, NCT02717884, NCT02261779, and NCT02273102). It remains to be seen whether LSD1 inhibitors will have efficacy in patients with *MLL-r* leukemias.

### Polycomb Protein Inhibitors

Polycomb repressive complexes 1 and 2 are two protein complexes involved in chromatin modulation and transcriptional repression. Both have been implicated in *MLL-r* leukemias. PRC1 contains core components BMI1, RING2A, and RING2B and mediates H2Ak119 monoubiquitination. Several groups have investigated the functional requirement of these PRC1 components in *MLLr* leukemia. Initial reports investigating the role of BMI1 using BMI1 knockdown and/or MLL-AF9 leukemias generated on a *Bmi1*^−^*^/^*^−^ background suggested that PRC1 canonical function is not required for *MLLr* leukemogenesis, although some transcriptional and minor functional effects on leukemia initiating cell frequency were observed (66, 217, 218). In contrast, combined knockout of *Ring1a/b* in a murine model of MLL-AF9-induced leukemia was not tolerated (219). The stark discrepancy in phenotypic consequences between knockout of different PRC1 components have not been well resolved and may relate to differently composed subcomplexes and/or functions outside of canonical PCR1.

In addition, the non-canonical PRC1 member CBX8 has been implicated leukemogenesis particularly mediated by MLL-AF9 and MLL-ENL. As mentioned earlier, AF9 and ENL bind CBX8 (63, 64), and leukemia initiation and maintenance in murine models MLL-AF9 and MLL-ENL AML were dependent on CBX8 (66). CBX function in MLLr leukemia appears to be independent of its role in PRC1, however, and instead involve the recruitment of the histone acetyl transferase Tip60 to fusion target loci (66). This is further supported by the finding that binding of CBX8 to ENL reverses the repressor activity of CBX8 (220). In mouse models, deletion of CBX8 had no detrimental effect on normal hematopoiesis, suggesting CBX8 and/or Tip60 could be interesting target for future development of inhibitors.

PRC2 consists of the canonical components EZH2, EED, and SUZ12. EZH2 is a histone methyltransferase targeting H3K27. Multiple groups have documented decreased proliferation, differentiation, and loss of stem cell potential in *MLL* fusion leukemias when any component of the complex was genetically knocked down or deleted (215, 221–223). This effect was most prominent with depletion of the universal components of the complex, EED or SUZ12, whereas EZH1 and EZH2 have somewhat redundant functions (215, 221). Impairment of the leukemia phenotype was largely attributable to de-repression of INK4A and ARF, although deregulation of other PRC2 target genes such as GATA2 and EGR1 were also implicated (223). Two inhibitors (DZNep and UNC1999) have shown efficacy *in vitro* against *MLL-r* leukemias and have prolonged survival in xenograft models of disease (224, 225). Another small molecule inhibitor, which disrupts the protein–protein interaction between EZH2 and EED, has had similar utility in MLL-AF9 models without any effect on non-transformed cells (222). However, inactivating mutations in PRC2 components has also been observed in AML and MDS patients and at least in MDS correlates with poor prognosis. As an added wrinkle to the story, the observation that AF10, a necessary cofactor for H3K79 di- and trimethylation by DOT1L, binds unmodified H3K27 (72) suggests that inhibition of EZH2 or PRC2 components could also facilitate H3K79 methylation on MLL-fusion target genes, although there is currently no experimental data to document that this is the case. Pharmacologic inhibitors of EZH2 are in clinical trials for diseases where PRC2/EZH2 hyperfunction is clearly linked to malignant transformation (such as lymphomas with activating EZH2 mutations or INI1-negative solid tumors). EZH2 inhibition is not currently investigated in clinical trials for AML or *MLL-r* leukemias.

### Agents that Counteract Antiapoptotic Mechanisms

As previously mentioned, *MLL-r* leukemias have been reported to be resistant to programmed cell death (86–89). Leukemias with t(4,11) translocations (*MLL-AF4*) tend to have elevated levels of prosurvival BCL-2 protein, which counteracts the intrinsic mitochondria-mediated apoptotic pathway (89). *In vitro* cytotoxicity studies of the pan-BCL-2 family inhibitor obatoclax demonstrated efficacy of this agent against a panel of *MLL-r* infant leukemias as well as *MLL-r* cell lines (165, 226); obatoclax also synergized with multiple standard chemotherapeutic agents (226). Recent work has suggested that t(4,11) leukemias tend to have highest expression of BCL-2 of multiple classes of acute leukemias and that the MLL-AF4 protein upregulates BCL-2 expression *via* DOT1L-mediated H3K79 methylation (92). The selective BCL-2 inhibitor, ABT199 (venetoclax), which has shown promise in clinical trials against chronic lymphocytic leukemia and other hematopoietic malignancies (227–229), was effective both *in vitro* and in xenograft models against *MLL-r* leukemias in combination with cytotoxic chemotherapies and with a DOT1L inhibitor. Further work in xenograft models confirms not only the enhanced sensitivity of *MLL-r* leukemias to BCL-2 inhibition compared to other subgroups of ALL but also the enhanced efficacy of combined inhibition of BCL-2 and BCL-XL (230). These data suggest yet another class of targeted agents that may prove useful adjuncts to therapy in *MLL-r* leukemias.

### Cell Cycle Checkpoint Inhibitors

Recent studies have identified cyclin-dependent kinase 6 (CDK6) as a target gene of MLL fusion proteins (231). CDK6 binds to D cyclins and promotes cell cycle progression through phosphorylation and inhibition of target genes such as RB1. *MLL-r* leukemias seem to be dependent on CDK6, but not on CDK4, for growth and proliferation (231, 232). This dependence on CDK6 was not seen in non-*MLL-r* leukemias. Furthermore, treatment of either *MLL-r* cell lines or primary patient AML cells with the CDK6 inhibitor palbociclib (PD0332991) led to both growth inhibition, decreased colony formation, and a differentiated phenotype (232). Third-generation transplant recipient mice given palbociclib-treated *MLL-AF9* cells had decreased disease burden and prolonged survival compared to mice given *MLL-AF9* control cells. These preclinical data suggest that CDK6 is a potential target for *MLL-r* leukemias; accordingly, there is an active phase Ib/IIa clinical trial out of the University of Ulm (NCT02310243) of palbociclib as monotherapy for adults with *MLL-r* leukemias.

### Menin Inhibitors

As mentioned earlier, the protein Menin interacts with the *N*-terminal portion of the MLL1 protein and has been shown to be essential for MLL fusion protein leukemogenesis [(6–9); see also Figure 2]. Menin also interacts with wild-type MLL1, and studies in mice have shown that genetic deletion of Menin affects long-term hematopoietic stem cell potential and B-lineage lymphoid progenitors (233). The therapeutic window of Menin inhibition for *MLL-r* leukemias is therefore uncertain. Nonetheless, several groups have developed small molecule inhibitors that disrupt the interaction between MLL1 and Menin and have shown *in vitro* and *in vivo* impairment of leukemia growth and proliferation, irrespective of the MLL fusion partner (234–237). As these studies were all short-term experiments with murine models of *MLL-r* leukemias, longer-term preclinical models will be essential studies to perform before development of clinical trials with these agents.

### Dinaciclib

Due to the association of common *MLL* fusion partners in the SEC with pTEFb, the role of specific pTEFb inhibitors has also been examined as potentially useful in targeting *MLL-r* leukemias (Figure 2). The efficacy of the CDK9 inhibitor (part of the pTEFb complex) Flavopiridol on *MLL-r* leukemia cells has long been recognized (46). Dinaciclib, which inhibits the CDK9 component of pTEFb, showed efficacy in preclinical models both *in vitro* and *in vivo*, inducing apoptotic cell death in *MLL-r* leukemia models and inhibition of MLL target genes (165, 238). No toxicity data were reported in these studies, so it remains to be seen whether this inhibitor will demonstrate appropriate specificity for MLL target genes without causing inordinate toxicity due to global repression of RNA polymerase II.

## Final Thoughts

*MLL* translocations lead to aberrant expression of stem cell genetic programs in hematopoietic cells, which leads to a particularly aggressive subtype of leukemias in children and adults. Outcomes with conventional chemotherapy remain suboptimal to dismal, and hematopoietic stem cell transplantation has not proven to be beneficial except in the most high-risk infant patients. Despite extensive resources and manpower devoted to a better understanding of MLL fusion biology, we still possess an inadequate understanding of the pathophysiology of this disease. Accompanying the ever increasing number of fusion partners identified is the ever widening circle of epigenetic regulators thought to be involved in genetic dysregulation upon expression of *MLL* translocations. However, in the era of targeted therapies, we may finally be at the cusp of discovering combinations of therapeutic agents that can improve the outcomes for these patients.

## Author Contributions

Both authors conducted extensive literature review and co-wrote the final manuscript.

## Conflict of Interest Statement

KB: I hold two patents with respect to the DOT1L inhibitor discussed in this manuscript (pertaining to it’s use in leukemias with high MN1 expression, and pertaining to the role of MDR1 mediated drug resistance). Neither is directly relevant to the discussion in this review article. My husband has just accepted a job as a medical director at Janssen. His work will not involve any of the agents discussed in this review article. AW declare that the research was conducted in the absence of any commercial or financial relationships that could be construed as a potential conflict of interest.
